# High Presence of Extracellular Hemoglobin in the Periventricular White Matter Following Preterm Intraventricular Hemorrhage

**DOI:** 10.3389/fphys.2016.00330

**Published:** 2016-08-03

**Authors:** David Ley, Olga Romantsik, Suvi Vallius, Kristbjörg Sveinsdóttir, Snjolaug Sveinsdóttir, Alex A. Agyemang, Maria Baumgarten, Matthias Mörgelin, Nataliya Lutay, Matteo Bruschettini, Bo Holmqvist, Magnus Gram

**Affiliations:** ^1^Department of Clinical Sciences Lund, Pediatrics, Lund University, Skane University Hospital Lund, Sweden; ^2^Department of Clinical Sciences Lund, Infection Medicine, Lund University, Skane University Hospital Lund, Sweden; ^3^ImaGene-iT AB Lund, Sweden

**Keywords:** intraventricular hemorrhage, hemoglobin, periventricular white matter, immature brain, plasticity, peroxidase activity, imaging

## Abstract

Severe cerebral intraventricular hemorrhage (IVH) in preterm infants continues to be a major clinical problem, occurring in about 15–20% of very preterm infants. In contrast to other brain lesions the incidence of IVH has not been reduced over the last decade, but actually slightly increased. Currently over 50% of surviving infants develop post-hemorrhagic ventricular dilatation and about 35% develop severe neurological impairment, mainly cerebral palsy and intellectual disability. To date there is no therapy available to prevent infants from developing either hydrocephalus or serious neurological disability. It is known that blood rapidly accumulates within the ventricles following IVH and this leads to disruption of normal anatomy and increased local pressure. However, the molecular mechanisms causing brain injury following IVH are incompletely understood. We propose that extracellular hemoglobin is central in the pathophysiology of periventricular white matter damage following IVH. Using a preterm rabbit pup model of IVH the distribution of extracellular hemoglobin was characterized at 72 h following hemorrhage. Evaluation of histology, histochemistry, hemoglobin immunolabeling and scanning electron microscopy revealed presence of extensive amounts of extracellular hemoglobin, i.e., not retained within erythrocytes, in the periventricular white matter, widely distributed throughout the brain. Furthermore, double immunolabeling together with the migration and differentiation markers polysialic acid neural cell adhesion molecule (PSA-NCAM) demonstrates that a significant proportion of the extracellular hemoglobin is distributed in areas of the periventricular white matter with high extracellular plasticity. In conclusion, these findings support that extracellular hemoglobin may contribute to the pathophysiological processes that cause irreversible damage to the immature brain following IVH.

## Introduction

Cerebral intraventricular hemorrhage (IVH) was described for the first time as a post-mortem diagnosis (Larroche, 1964) and was assumed to be a fatal event. Although modern perinatal medicine has led to a significant decrease in the overall incidence of IVH in preterm infants, i.e., from 50% in the late 1970s to the current 15–25% (Philip et al., 1989; Horbar et al., 2002; Hamrick et al., 2004), it continues to be an important problem in the neonatal intensive care unit as a consequence of increased survival of extremely preterm infants (born before 28 gestational weeks; Group et al., 2009; Ishii et al., 2013). To date, the incidence of IVH reaches approximately 45% in premature infants with birth weight less than 750 g (Wilson-Costello et al., 2005). Approximately 50–75% of the preterm infant survivors with high grade IVH develop neurodevelopmental disability such as cerebral palsy, intellectual disability, and/or post-hemorrhagic ventricular dilatation (PHVD; Luu et al., 2009). Hence, IVH and its resultant neurologic and psychiatric sequelae continue be an important public health concern worldwide.

The etiology of IVH is multifactorial, complex and heterogeneous. It is believed that an inherent fragility of the immature germinal matrix vasculature sets the ground for hemorrhage. The germinal matrix lies within an arterial end zone, and it is directly connected to the deep galenic venous system (Pape and Wigglesworth, 1979; Nakamura et al., 1990) thereby exposing the germinal matrix to insults of arterial ischemia-reperfusion and to venous congestion (Takashima and Tanaka, 1978; Pape and Wigglesworth, 1979). It has been shown in preterm infants with IVH, that deposition of extravasated blood in the intraventricular space is followed by lysis of the red blood cells (RBC) resulting in a subsequent release of extracellular hemoglobin (Hb) into the cerebrospinal fluid (CSF; Gram et al., 2013). Once Hb escapes the intra-erythrocyte compartment, it is highly reactive and rapidly oxidized from ferrous (Fe^2+^, denoted oxyHb) to ferric (Fe^3+^, denoted metHb) Hb (Umbreit, 2007; Gram et al., 2013), which, readily releases the heme group (Bunn and Jandl, 1968). Free heme is very redox reactive and can damage lipids, proteins and DNA through oxidative modification, cross-linking and fragmentation (Kumar and Bandyopadhyay, 2005). Moreover, heme is also highly lipophilic and binds to lipids intercalating into cell membranes, which results in toxic cytolytic effects through both oxidative and non-oxidative mechanisms (Wagener et al., 2001). In the context of adult intracranial hemorrhage (ICH) it is widely recognized that blood and extracellular Hb activate cytotoxic, oxidative and inflammatory pathways, eventually leading to tissue damage (Xi et al., 2006; Fang et al., 2013). Using a preterm rabbit pup model of IVH, we recently showed that extracellular Hb leads to structural damage of the choroid plexus ependyma at 24 h after IVH and cause severe cellular disintegration with loss of normal villous morphology and signs of cellular apoptosis/necrosis at 72 h after IVH (Gram et al., 2013, 2014). Importantly, in preterm infants, the breakdown of the blood-brain barrier (BBB) have been suggested to be a key event behind the subsequent periventricular white matter damage following IVH (Volpe, 2009).

We hypothesized that following hemorrhage, the ventricular lining is breached and substantial amounts of RBCs and released extracellular Hb, i.e., not retained within the RBCs, may pass over the barrier to enter into periventricular brain areas. Here we report that following IVH, in the preterm rabbit pup, high amounts of extracellular Hb are widely distributed within the periventricular white matter, predominantly localized to areas with high mobility and permeability.

## Materials and methods

### Animals

The animal protocols were approved by the Swedish Animal Ethics Committee in Lund. We used the well-established preterm rabbit pup model of glycerol-induced IVH in accordance with previous description (Sveinsdottir et al., 2012). Briefly, the experiments were performed on a total of 47 rabbit pups from 23 litters delivered at gestational day 29 (term 32 days). A half-breed between New Zealand White and Lop was used. The pups were delivered by cesarean section after the does were anesthetized with i.v. propofol (5 mg/kg) and with local infiltration of the abdominal wall using lidocaine with adrenaline (10 mg/ml + 5 μl/ml, 20–30 ml). After birth the pups were dried, weighed and placed in an infant incubator with a temperature of 34–35°C and ambient humidity (created by the placement of a water container within the incubator). At 2–3 h of age the pups were hand-fed with 2 ml (100 ml/kg/day) of cat milk formula (KMR; PETAG Inc, USA) using 3.5 French feeding tube, thereafter every 12 h, increasing the meal by 1 ml every 24 h. At approximately 3 h of age, the pups received an i.p. injection of 50% (v/v) sterile glycerol (6.5 g/kg; Teknova, Hollister, CA, USA) to induce IVH. Ultrasound imaging of the brain was performed at 6 h of age to detect and grade IVH and after that at 24, 48, and 72 h of age using the VisualSonics Vevo 2100 (VisualSonics Inc., ON, Canada) with a MS-550D 40 MHz transducer. Only animals with a large IVH at 6 h were used for data analysis and animals with no detectable IVH at all time-points on cranial ultrasound were used as controls. Measurements of ventricular size for assessment of PHVD were obtained at the level of the midseptal nucleus in a coronal view at 6, 24, 48, and 72 h of age. Each ventricle was measured horizontally from the midbrain plane to the lateral wall of the ventricle. Reproducibility and accuracy of ventricular measurements in this animal model using high-frequency ultrasound have been described previously (Sveinsdottir et al., 2012). The animals were euthanized at 72 h and the brains were prepared as described below.

### Tissue collection and processing

For histochemistry, immunolabeling and SEM, rabbit pups were euthanized at 72 h of age (IVH *n* = 6; sham control *n* = 6) by isoflurane anesthesia followed by saline and freshly prepared 4% paraformaldehyde (PFA) perfusion. Afterwards the brains were dissected out from the skulls and were immersed in 4% PFA. A change to fresh PFA was done after 3–6 h and brains were immersed for a total of 24 h, at 4°C. Brains were then cryo-protected by sequential immersion in 15% sucrose (diluted in phosphate buffer saline, PBS, 0.1 M, pH 7.4) for 6 h and in 25% sucrose (diluted in PBS) for another 6 h. Brains were mounted in TissueTec (Sakura Finetek, Torrance, CA, USA) and frozen (at around −60°C) in cryomolds, on dry ice in isopentane (2-methylbutane, Sigma-Aldrich, St. Louis, MO, USA). Sections (12μm) were cut on a cryotome (Microm, HM 500 OM, Microm Laborgeraete GmbH, Walldorf, Germany). Sections, were collected on SuperFrost plus slides (Menzel, Braunschweig, Germany), two per slide, starting from the end of the olfactory bulb to the end of the midbrain (a detailed description of the areas analyzed can be found in the results section).

Sections were stored at −20°C until used for the labeling (as described below).

### Hematoxylin-eosin

To define the brain neuroanatomy and thereby enable selection of the corresponding levels from all animals used for the histochemical and immunolabeling analysis (below), every 10–15th section was stained with HE. The HE staining procedure was performed as follows: Sections were air-dried, at room temperature (RT) or 37°C, for 20–30 min, rinsed in PBS, 2 × 5 min, followed by a rinse in dH_2_O for 1 min. Sections were immersed in Mayers Hematoxylin (Histolab, Gothenburg, Sweden), for 2 min, followed by three fast 1 min rinses in dH_2_O. Sections were then immersed in sodium bicarbonate (0.1%), for 1 min followed by immersion in dH_2_O for 2 × 1 min. Sections were immersed in 70% ethanol for 2 min, in Eosin (Histolab, 0.2% diluted in 70% ethanol acidified with glacial acetic acid) for 3 min. Sections were dehydrated in alcohol (96% x2 and 100% x2 for 3 min in each solution), and in xylene (100% for >10 min). Sections were mounted in Pertex (Histolab) and cover slipped.

### Peroxidase histochemistry

To detect PO, and increase thereof by Hb, and to be able to determine its distribution in the brains of all animal groups we performed an adapted protocol of the enhanced peroxidase reaction of cryosections (Strum and Karnovsky, 1970).

About every 10th section was stained for PO, in parallel sections to HE and immunolabeled sections (see below), containing the selected ROI's at the corresponding levels. Briefly, sections were air-dried, at RT or 37°C, for 20–30 min, rinsed in PBS 2 × 10 min. The peroxidase reaction was performed in a solution containing 3,3′-diaminobenzidine (DAB, 0.5 mg/ml diluted in PBS, Sigma-Aldrich) containing 0.015% H_2_O_2_ (Merck, USA), for 10 min at RT. Sections were then rinsed in PBS 3 × 5 min, and in H_2_O for 1 min. Counterstaining was performed with hematoxylin (HTX, Mayers, Histolab), via immersion of sections in HTX for 2 min, and rinses in dH_2_O followed for 3 × 1 min. Sections were then dehydrated in alcohol (70% for 1 min, 96% for 2 × 5 min and 100% for 2 × 7 min), and in xylene (100% for 2 × 5 min). Sections were mounted in Pertex (Histolab) and cover slipped.

As controls for the PO staining, sections were pre-incubated in H_2_O_2_ (0.03%, i.e., peroxidase quenching) for 10 min at RT, which totally quenched the endogenous erythrocyte and periventricular tissue peroxidase reaction in non-IVH animals.

### Immunofluorescence labeling

To specifically detect Hb, and as a comparison with PO, we performed single immunofluorescence labeling of Hb, of selected ROI's. Double immunofluorescence with PSA-NCAM was performed to further elucidate the indicated distribution of Hb in differentiation zones and regions of neural plasticity.

The protocol used for single and double immunofluorescence labeling was as follows: Sections were air-dried at RT or 37°C. Sections were encircled with silicon (PAP-pen) and rinsed in PBS 2 × 5 min. Sections were incubated (always in moisture chamber) with 1% BSA (diluted in PBS containing 0.05% TX, denoted PBSTXBSA) for 60 min, at RT. Sections were then incubated in one primary antibody or in a mixture of primary antibodies (“cocktail”) diluted in PBSTXBSA overnight, at 4°C. Adjacent sections were incubated with only PBSTXBSA, as controls for the primary and secondary antibodies.

Primary antibodies used were against Hb (diluted 1:750, goat IgG from GenWay Biotech, GWB-F26D80) and PSA-NCAM (diluted 1:2000, mouse IgM from Merck Millipore, USA, MAB5324). Sections were rinsed in PBS for 3 × 3 min and were incubated in one secondary antibody or in a mixture of secondary antibodies (diluted 1:200 in PBSTXBSA) for 60 min, at RT.

Secondary antibodies used (AffiniPure Fab2 for multilabeling) were made in donkey against mouse IgM conjugated with AF488 or goat IgG conjugated with Rhodamine Red (all from Jackson ImmunoResearch, West Grove, PA, USA). Sections were then rinsed in PBS for 3 × 3 min, and incubated in DAPI (0.1μM, diluted in PBS, from Invitrogen, 62,247) for 30 min, at RT. Following rinses in PBS for 3 × 5 min, sections were mounted and cover slipped in Fluoroshield (Abcam, England, ab104135).

### Bright-field and fluorescence microscope analyses and data documentation

Microscope analyses were performed on a wide-field Olympus microscope (IX73) equipped for bright field and epi-fluorescence microscopy. Digital image documentation was performed with a DP80 camera (Olympus).

Slide scanning, used for overviews and some of the detailed histological and histochemical descriptions, were performed on a Hamamatsu NanoZoomer 2.0-HT Digital slide scanner: C10730. Scanning were performed with a 40 × magnification lens. Images used for illustrations, from ROI's, were grabbed with the viewer software NDP.view2 Viewing software.

### Scanning electron microscopy

To investigate if intact RBCs were present within the tissue, cryosections were analyzed at high magnification using SEM. Cryosections from IVH and sham control brains containing the selected ROI's were air-dried at RT, rinsed 2 × 5 min in PBS, and in H_2_O for 1 min. Specimens were then fixed over night at RT with 2.5% glutaraldehyde in 150 mM cacodylate buffer. They were washed with cacodylate buffer and dehydrated with an ascending ethanol series from 50% (v/v) to absolute ethanol.

The specimens were then subjected to critical point drying with carbon dioxide and absolute ethanol was used as an intermediate solvent. Sections were mounted on aluminum holders, sputtered with 20 nm palladium/gold and examined in a Philips/FEI XL 30 FESEM scanning electron microscope using an Everhart-Tornley secondary electron detector. Image processing was done with the Scandium software for simple image acquiring and auto-storage into the Scandium database.

Electron microscopic analysis was performed at the Core Facility for Integrated Microscopy (Panum Institute, University of Copenhagen, Denmark).

## Results

### Histochemistry, immunofluorescence, and neuroanatomical description

Sections from IVH and sham control animals, at the same levels (see below), were stained for hematoxylin and eosin (HE) and peroxidase activity of Hb (PO, for detailed description see below). Parallel sections were single immunofluorescence labeled for Hb, or double immunofluorescence labeled for Hb and polysialic acid neural cell adhesion molecule (PSA-NCAM). Furthermore, parallel sections were analyzed with scanning electron microscopy (SEM).

From the HE, PO staining and Hb immunolabeling a number of subventricular and periventricular anatomically comparable regions of interests (ROI) were chosen. Neuroanatomically defined ROI's were located in the rostral forebrain (Level 1), caudal forebrain (Level 2), rostral midbrain (Level 3) and caudal midbrain (Level 4). The definition of neuroanatomy and nomenclature used follows that described in the “Atlas of the rabbit brain and spinal cord,” by Shek et al. (1986). A detailed neuroanatomical description of the ROI's can be found in Supplementary Material.

### Hemorrhage distribution following preterm rabbit pup IVH

**Figure 1:**
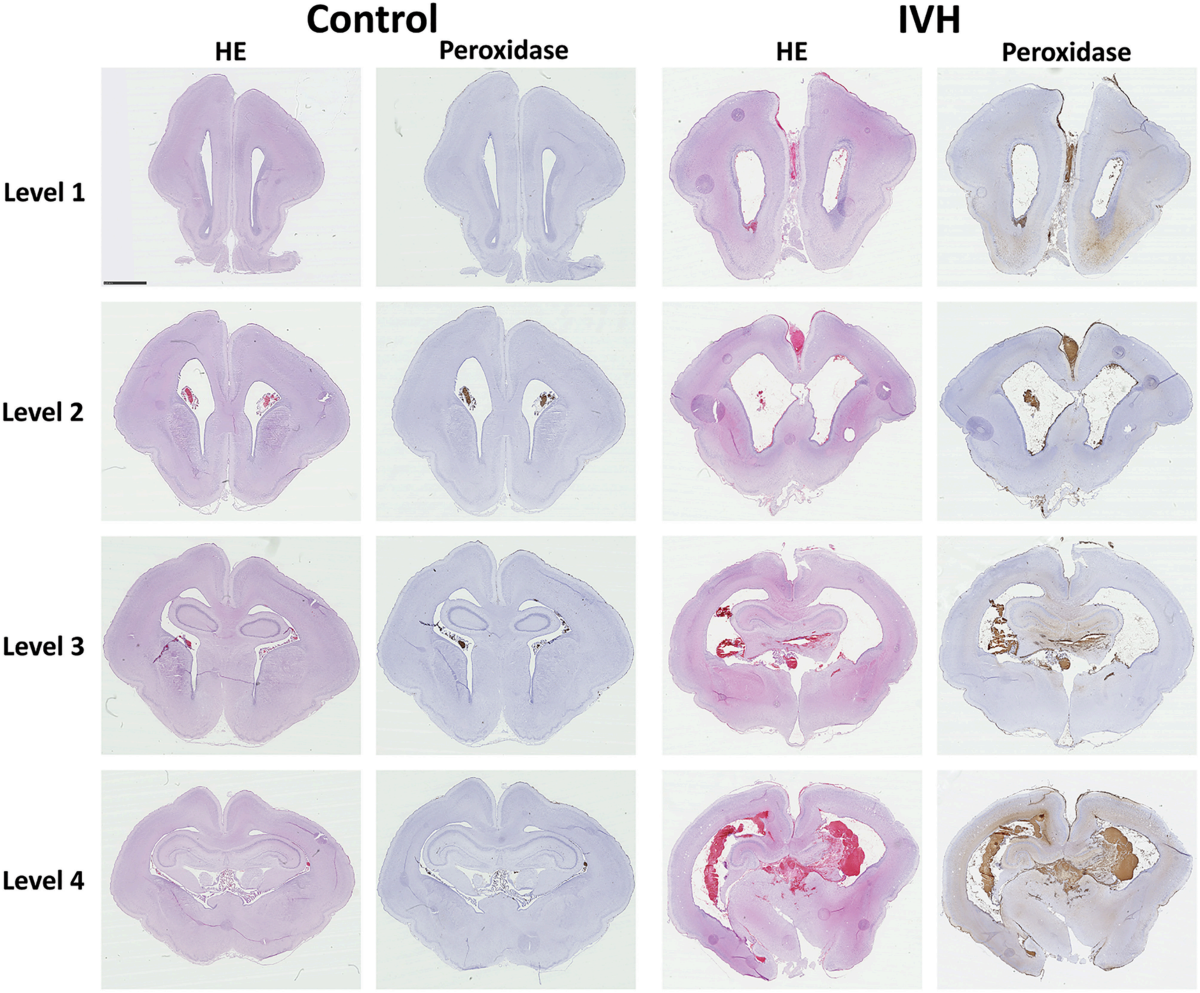
**Hemorrhage distribution following preterm rabbit pup IVH**. Following IVH in preterm rabbit pups, the distribution of the hemorrhage was investigated. Rabbit pups with IVH or sham controls were euthanized at 72 h of age followed by saline and freshly prepared 4% PFA perfusion. Afterwards the brains were dissected out from the skulls and immersed in 4% PFA. Brains were prepared and sections from Control and IVH animals, at the levels of rostral forebrain (Level 1), caudal forebrain (Level 2), rostral midbrain (Level 3) and caudal midbrain (Level 4), were stained for hematoxylin and eosin (HE) and peroxidase activity of Hb (Peroxidase) as described in the Materials and Methods Section. Microscope analyses were performed on a wide-field Olympus microscope (IX73) and slide scanning were performed on a Hamamatsu NanoZoomer 2.0-HT Digital slide scanner: C10730. Scanning was performed with a 40 × magnification lens. Scale bar indicate 2.5 mm and is applicable for all images.

Following IVH in preterm rabbit pups, the distribution of the hemorrhage was investigated at 72 h of age. Erythrocytes (HE stained) displayed a widespread distribution of the bleeding throughout all evaluated parts of the brain, i.e., from frontal parts in the rostral forebrain (Level 1) to posterior parts at the caudal midbrain (Level 4) (Figure 1, HE). A large ventricular dilation was seen in the IVH animals, not present in the sham controls, in congruence with previous studies (Sveinsdottir et al., 2012). Although bleeding, in form of intact blood cells, was found throughout all evaluated parts of the brain it was most prominent at the more occipital parts, i.e., rostral midbrain (Level 3) and caudal midbrain (Level 4). In these parts of the brain, a high amount of RBCs was observed within the heavily distended choroid plexus, the subfornical organ (SFO), one of the three sensory circumventricular organs (Cottrell and Ferguson, 2004), and also to some degree within the hippocampus and dorsal thalamus. In the more frontal parts, i.e., in the rostral forebrain (Level 1) and caudal forebrain (Level 2), the blood cells were most obvious within the ventricle and also in the subventricular zone (SVZ) (Figure 1, Level 1 and 2). Analysis of the ventricular ependymal integrity showed some areas that were disrupted, often associated with periventricular infiltrating blood cells, including macrophages.

### Extracellular Hb is found widespread within the periventricular white matter

**Figure 2:**
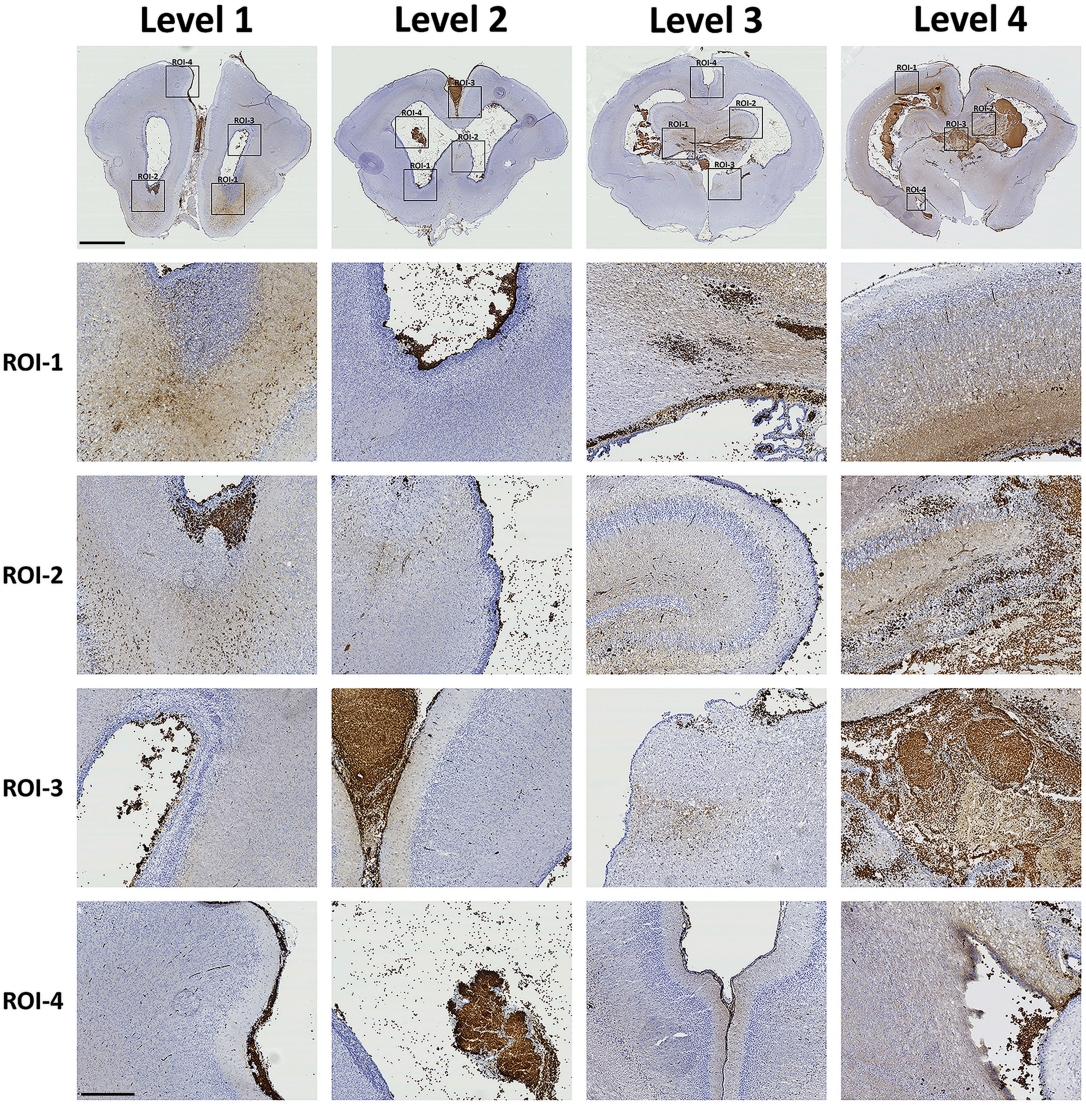
**Characterization of Hb following preterm rabbit pup IVH**. The presence of Hb following IVH was characterized within the brain utilizing the inherent peroxidase activity of Hb. Rabbit pups with IVH or sham controls were euthanized at 72 h of age followed by saline and freshly prepared 4% PFA perfusion. Afterwards the brains were dissected out from the skulls and immersed in 4% PFA. Brains were prepared and sections and a number of subventricular and periventricular anatomically comparable regions of interests (ROI) were chosen. Neuroanatomically defined ROI's located in the rostral forebrain at the levels of rostral forebrain (Level 1), caudal forebrain (Level 2), rostral midbrain (Level 3) and caudal midbrain (Level 4), were stained for peroxidase activity of Hb as described in the Materials and Methods Section. Microscope analyses were performed on a wide-field Olympus microscope (IX73) and slide scanning were performed on a Hamamatsu NanoZoomer 2.0-HT Digital slide scanner: C10730. Scanning was performed with a 40 × magnification lens. Images used for illustrations, from regions of interest (ROI), were grabbed with the viewer software NDP.view2 Viewing software. Scale bar of slide scan image indicate 2.5 mm and of ROI images indicate 500 μm.

The presence of Hb following IVH was characterized within the brain utilizing the inherent peroxidase activity of Hb (Cooper et al., 2008; Kapralov et al., 2009). Thus, by using a modified protocol of an intensified PO (Strum and Karnovsky, 1970) in rabbit pup brain cryosections, we evaluated the distribution of Hb in all experimental groups. The specificity was tested against Hb immunolabeling (see below). PO histochemical results showed a high amount of PO activity widely distributed throughout the brain, from the rostral forebrain (Level 1) through the caudal forebrain (Level 2), rostral midbrain (Level 3) and into the caudal midbrain (Level 4) (Figure 1, Peroxidase). Corresponding to the Hb immunolabeling (see below) and HE staining, a relatively high PO activity was observed in the ventricular and more posterior parts of the brain. PO activity was found in all areas stained positive for erythrocytes and displayed an intense brown color (Figure 2). Corresponding to the HE staining, the PO activity related to RBCs was observed mainly within the ventricle (Level 1, ROI-3 and Level 2, ROI-1 and ROI-3), in the SVZ (Level 1, ROI-2), the choroid plexus (Level 2, ROI-4), the SFO (Level 4, ROI-3) and in some rare cases within the parenchyma of the rostral midbrain (Level 3, ROI-1) and caudal midbrain (Level 4, ROI-2) (Figure 2). Noticeably, in addition to intense labeling of erythrocytes, the PO staining also produced a more diffuse brighter brown color, i.e., not associated with RBCs, that was widely distributed within the brain parenchyma in periventricular brain areas. Consistent with the HE and RBC related PO activity, the most extensive bright PO activity was observed in the more posterior part of the midbrain, in the (but not restricted to) dorsal ventricle and cortex cerebri (Level 4, ROI-1), corpus callosum (Level 3, ROI-1), hippocampus (Level 4, ROI-2 and Level 3, ROI-2), thalamus and SFO (Level 4, ROI-3). However, clearly different from the HE and RBC related PO activity, the bright PO activity, displayed a relatively high staining also in the more anterior parts of the brain. Moreover, it was found widely distributed throughout the brain, not only from anterior to posterior parts, but also very clearly laterally out from the ventricle. Importantly though, it seemed as if the bright PO activity, was mostly located in areas corresponding to periventricular white matter.

**Figure 3:**
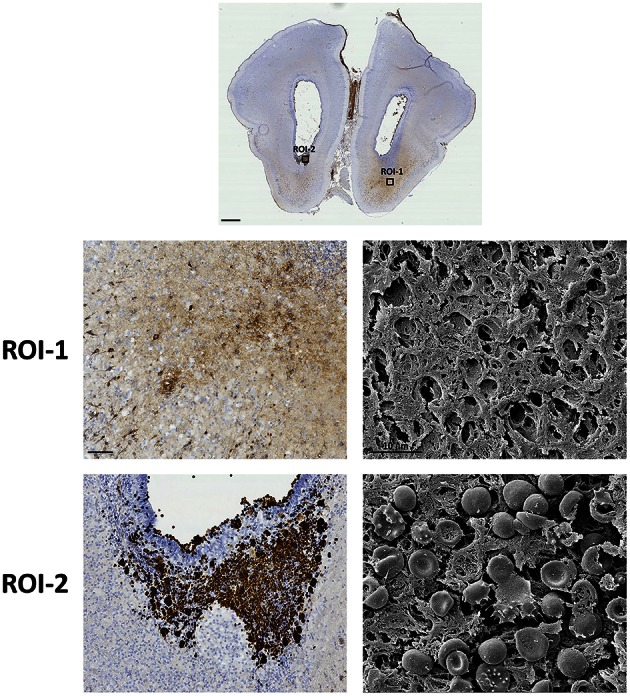
**Presence of intracellular and extracellular Hb**. The presence of RBCs and Hb following IVH was characterized within the brain utilizing the inherent peroxidase activity of Hb in combination with SEM. Rabbit pups with IVH or sham controls were euthanized at 72 h of age followed by saline and freshly prepared 4% PFA perfusion. Afterwards the brains were dissected out from the skulls and immersed in 4% PFA. Brains were prepared and sections at the levels of rostral forebrain (Level 1) were stained for peroxidase activity of Hb or prepared for high magnification SEM as described in the Materials and Methods section. Bright-field microscope analyses were performed on a wide-field Olympus microscope (IX73) and slide scanning were performed on a Hamamatsu NanoZoomer 2.0-HT Digital slide scanner: C10730. Scanning was performed with a 40 × magnification lens. Images used for illustrations, from regions of interest (ROI), were grabbed with the viewer software NDP.view2 Viewing software. Scale bar of slide scan image indicate 1.0 mm and of ROI images indicate 100 μm. SEM specimens were examined in a Philips/FEI XL 30 FESEM scanning electron microscope using an Everhart-Tornley secondary electron detector. Image processing was done with the Scandium software for simple image acquiring and auto-storage into the Scandium database. Scale bar of SEM images indicate 10 μm.

In order to evaluate if intact RBCs were present and consequently could be responsible for the PO activity found in the intense vs. bright areas, SEM was used (Figure 3). Results showed that in areas with the most intense cellular PO activity, intact RBCs are present (Figure 3, ROI-2) whereas in areas of less intense PO activity, no intact RBCs are present (Figure 3, ROI-1). Thus, non-cellular PO activity most likely corresponded to extracellular Hb.

**Figure 4:**
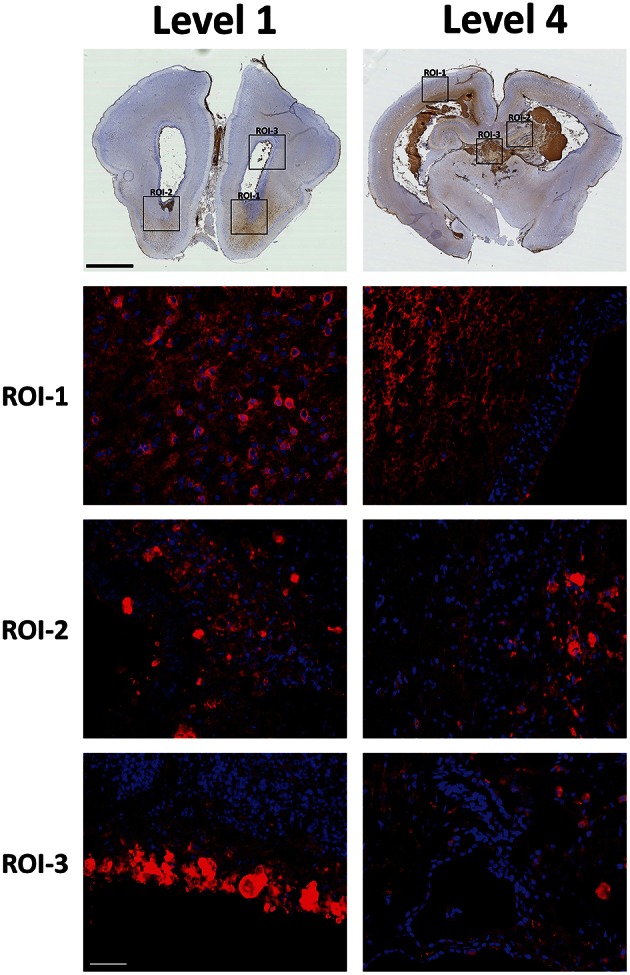
**Hb immunolabeling within the brain following preterm rabbit pup IVH**. The presence of Hb following IVH was characterized by Hb (displayed as red) immunolabeling of selected regions of interests (ROI) of peroxidase activity corresponding areas, recorded in parallel sections. Rabbit pups with IVH or sham controls were euthanized at 72 h of age followed by saline and freshly prepared 4% PFA perfusion. Afterwards the brains were dissected out from the skulls and immersed in 4% PFA. Brains were prepared and sections at the levels of rostral forebrain (Level 1) and caudal midbrain (Level 4), were immunolabeled for Hb as described in the Materials and Methods Section. Fluorescence microscope analyses were performed on a wide-field Olympus microscope (IX73) and slide scanning were performed on a Hamamatsu NanoZoomer 2.0-HT Digital slide scanner: C10730. Scanning was performed with a 40 × magnification lens. Images used for illustrations, from ROI's, were grabbed with the viewer software NDP.view2 Viewing software. DAPI labeled cell nuclei are blue. Scale bar of slide scan images indicate 2.5 mm and of ROI images indicate 25 μm.

To finally confirm that the recorded PO activity was in fact related to the presence of Hb we conducted Hb immunolabeling of corresponding areas, recorded in parallel sections (Figure 4). Results show that PO activity and Hb immunoreactivity respectively were present in corresponding periventricular white matter areas, in fiber tracts and accumulated in nucleated cells, thus defining the presence of extracellular Hb (Level 1, ROI-1 and -2 and Level 4, ROI-1-3). The specificity of Hb staining, shown by both PO activity and Hb immunolabeling, was further confirmed when neither PO staining nor Hb immunolabeling of control animals produced any labeling as compared to the IVH animals (Supplementary Figure 1).

**Figure 5:**
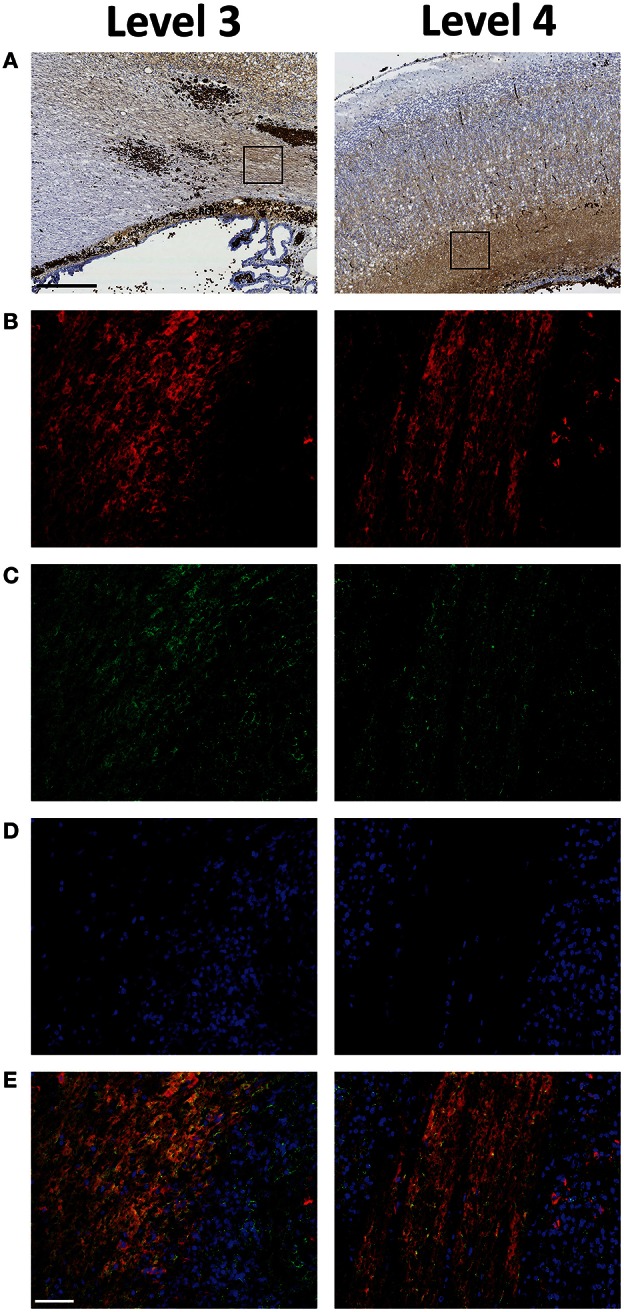
**Extracellular Hb is present within plastic areas of the periventricular white matter following preterm rabbit pup IVH**. The relation between Hb **(B)**, displayed in red and PSA-NCAM **(C)**, displayed in green, a marker of areas with high plasticity, characterized by double labeling of selected regions of interests (ROI) of peroxidase activity corresponding areas **(A)**, recorded in parallel sections. Rabbit pups with IVH or sham controls were euthanized at 72 h of age followed by saline and freshly prepared 4% PFA perfusion. Afterwards the brains were dissected from the skulls and immersed in 4% PFA. Brains were prepared and sections at the levels of rostral midbrain (Level 3) and caudal midbrain (Level 4), were immunolabeled for Hb and PSA-NCAM as described in the Materials and Methods section. DAPI labeled cell nuclei, displayed in blue, are shown in **(D)** and the merge of Hb, PSA-NCAM and DAPI is displayed in **(E)**. Fluorescence microscope analyses were performed on a wide-field Olympus microscope (IX73) and slide scanning were performed on a Hamamatsu NanoZoomer 2.0-HT Digital slide scanner: C10730. Scanning was performed with a 40 × magnification lens. Images used for illustrations, from ROI's, were grabbed with the viewer software NDP.view2 Viewing software. Scale bar of slide scan images indicate 500 μm and of ROI images indicate 25 μm.

The distribution of PO activity and Hb immunolabeling indicated presence of non-erythrocyte related Hb in cell bodies but also to a very large extent indicated presence in differentiation zones and axonal fiber tracts. Therefore, we investigated the relation between Hb immunolabeling and PSA-NCAM, a marker of areas with high plasticity (Nacher et al., 2002) such as that of the periventricular white matter of the immature brain (Luo et al., 2013) (Figure 5). Double labeling of Hb and PSA-NCAM displayed a high co-existence, thus clearly indicating that extracellular Hb is found within areas of high plasticity. Importantly, it should be noted that no difference in PSA-NCAM staining was recorded between the experimental groups (not shown).

## Discussion

This study, is to the best of our knowledge, the first to show that following IVH, in a preterm animal model, extracellular Hb, i.e., not retained within the erythrocytes, enters into and is widely dispersed via the periventricular white matter throughout wide areas of the immature brain. Furthermore, we show that a significant proportion of the extracellular Hb is distributed in areas of the periventricular white matter with high extracellular plasticity.

In the human preterm infant, hemorrhages restricted to the intraventricular space are believed to have their origin in ruptured vessels located within the germinal matrix with a subsequent extension of hemorrhage to the lateral ventricle (Ballabh, 2010). The IVH leads to a varying degree of PHVD which typically evolves over weeks. In the preterm rabbit pup model, IVH is invariably followed by PHVD which develops rapidly with a significant increase in ventricular size during the first 3 days (Sveinsdottir et al., 2012). In the current study, histological findings were recorded at 3 days of age and thus representative of IVH with a well-established PHVD. We have previously shown that ongoing hemolysis after IVH causes constant release of extracellular oxyHb in intraventricular CSF with the oxidized metabolite metHb reaching a peak at approximately 72 h (Gram et al., 2013). In previous studies we found that the metHb was a potent inducer of pro-inflammation, displaying a strong correlation with TNFα protein levels in intraventricular CSF and increased mRNA levels for TNFα, IL-1β and of HO-1 in periventricular brain tissue (Gram et al., 2013, 2014). Thus, our present observation of wide-spread presence of extracellular Hb in periventricular tissue at 72 h after IVH is consistent with our previous findings at the corresponding time-point.

In the current study, the inherent PO activity of Hb was utilized to identify and display the presence and distribution of both cellular and non-cellular retained Hb. The PO activity of Hb has previously mainly been utilized in plasma for qualitative or quantitative measurement of Hb (Grigorieva et al., 2013), however protocol for the use in histochemistry has been described (Strum and Karnovsky, 1970). The use of PO activity in tissue as an adjunct to specific Hb immunolabeling, which showed excellent co-distribution, for sensitive recognition of intracellular and extracellular Hb proved invaluable in the current study for achieving a spatial overview of the Hb distribution.

Specific staining for Hb, using PO activity and Hb immunolabeling, revealed presence of extracellular Hb within periventricular areas, such as the SVZ, with an increased gradient toward the less cellular dense white matter axonal tracts. These regions stained positively for PSA-NCAM signifying regions permissive for diffusion and mobility. In general, double labeling for Hb and PSA-NCAM exhibited a very high degree of co-existence. Of note, staining for extracellular Hb was frequently positive in periventricular areas adjacent to a morphologically intact ependyme. This suggests that extracellular Hb released during ongoing hemolysis within the intraventricular space diffuses passively through the ependyme driven by a concentration gradient. Alternatively, extracellular Hb has the potential to migrate in the white matter in directions with less restricted diffusion. Directional diffusion of small molecules, such as water, in axonal tracts has been extensively addressed using magnetic resonance imaging (MRI) technique (Le Bihan and Iima, 2015). To our knowledge, mobility within axonal tracts of molecules of similar size as Hb has not been addressed previously.

Preterm infants who develop IVH with PHVD are at increased risk for spastic diplegia, a well-defined sub-entity of cerebral palsy, with impaired motor function and spasticity predominantly present in the lower limbs (Linsell et al., 2016). The causal damage is considered to be located to the axons of the corticospinal tracts traveling in periventricular matter adjacent to the lateral ventricles. Altered periventricular white matter diffusion characteristics, as determined by MRI, were recently described in very preterm infants following IVH with PHVD (Brouwer et al., 2016). Extracellular Hb has been shown to cause oxidative damage to axonal myelin components and to cultured oligodendrocytes (Bamm et al., 2015). The present study shows that extracellular Hb is capable of reaching areas corresponding to those populated by the motor axons of the corticospinal tract. Preterm infants with IVH display reduced cortical gray matter volumes as determined by MRI segmentation (Vasileiadis et al., 2004). One explanation for this may be damage to glial progenitors in the SVZ causing a disturbed migration of supporting glial cells to developing cortical layers (Gressens et al., 1992). Extensive deposition of Hb in white matter may also lead to primary axonal damage leading to retrograde neuronal degeneration reflected as reduced cortical gray matter.

A striking and consistent finding, not previously reported in the literature, was the extensive hemorrhage detected in the region of the SFO. The SFO is located in the anterior wall of the third ventricle in near proximity to the foramen Monroi, connecting the third ventricle to the respective lateral ventricles. As one of the circumventricular organs, the SFO has a unique function as a sensory organ for blood osmolarity and plays an important role in regulation of fluid homeostasis and in cardiovascular regulation (Cottrell and Ferguson, 2004). IVH in the preterm rabbit pup is induced by intraperitoneal glycerol injection which causes an increase in serum osmolarity and thereby a pressure gradient over the BBB leading to vessel rupture (Conner et al., 1983). This is a reasonable analogy to the pathophysiological events leading to IVH in preterm infants where infusion of hyperosmolar solutions have been considered causal in occurrence of IVH (Papile et al., 1978). Continued study will evaluate the role of SFO hemorrhage as a possible primary event in the pathogenesis of IVH. Hemorrhage-induced damage to the sensory cells of the SFO may have clinically important long-term implications.

Extracellular Hb and its metabolites are well-documented initiators of oxidative stress and pro-inflammation (Xi et al., 2006; Fang et al., 2013). We propose extracellular Hb as a central up-stream initiator of mechanisms leading to irreversible damage following IVH in the developing brain. Therefore, the present findings are fundamental for furthered understanding of the causal relationship between extravasated blood primarily located within the intraventricular space and induction of mechanisms leading to cell and axonal damage in periventricular white matter, ultimately causing neurodevelopmental impairment. Development and investigations of tools of treatment to diminish Hb related damage during IVH may be one of the most important challenges.

## Author contributions

DL, SS, and MG designed the study. DL, OR, SV, KS, SS, AA, MB, MM, NL, MBR, BH, and MG acquired, analyzed and interpreted the data. DL, OR, SV, KS, SS, AA, MB, MM, NL, MBR, BH, and MG drafted and finally approved the content of this manuscript and are consequently in agreement to be accountable for all aspects of this work.

### Conflict of interest statement

The authors declare that the research was conducted in the absence of any commercial or financial relationships that could be construed as a potential conflict of interest. MG is co-founder of the company A1M Pharma AB. This does not present any conflict of interest.

## Abbreviations

BBB: Blood-brain-barrier
CSF: Cerebrospinal fluid
GM: Germinal matrix
Hb: Hemoglobin
HE: Hematoxylin and Eosin
HTX: Hematoxylin
ICH: Intracranial hemorrhage
IF: Immunofluorescence
IVH: Intraventricular hemorrhage
MetHb: Methemoglobin
MRI: Magnetic resonance imaging
OxyHb: Oxyhemoglobin
PFA: Paraformaldehyde
PHVD: Post-hemorrhagic ventricular dilatation
PO: Peroxidase activity
PSA-NCAM: Polysialic acid neural cell adhesion molecule
RBC: Red blood cells
ROI: Regions of interests
RT: Room temperature
SEM: Scanning electron microscopy
SFO: Subfornical organ
SVZ: Subventricular zone.
