# Corrigendum: High Presence of Extracellular Hemoglobin in the Periventricular White Matter Following Preterm Intraventricular Hemorrhage

**DOI:** 10.3389/fphys.2020.00027

**Published:** 2020-02-05

**Authors:** David Ley, Olga Romantsik, Suvi Vallius, Kristbjörg Sveinsdóttir, Snjolaug Sveinsdóttir, Alex A. Agyemang, Maria Baumgarten, Matthias Mörgelin, Nataliya Lutay, Matteo Bruschettini, Bo Holmqvist, Magnus Gram

**Affiliations:** ^1^Department of Clinical Sciences Lund, Pediatrics, Lund University, Skane University Hospital, Lund, Sweden; ^2^Department of Clinical Sciences Lund, Infection Medicine, Lund University, Skane University Hospital, Lund, Sweden; ^3^ImaGene-iT AB, Lund, Sweden

**Keywords:** Intraventricular hemorrhage, hemoglobin, periventricular white matter, immature brain, plasticity, peroxidase activity, imaging

In the original article, we neglected to include the funder “**the Swedish Foundation for Strategic Research**,” “**RBP14-0055**” **to** “**Magnus Gram**.”

The authors apologize for this error and state that this does not change the scientific conclusions of the article in any way. The original article has been updated.

