# Supplementary material for: High Presence of Extracellular Hemoglobin in the Periventricular White Matter Following Preterm Intraventricular Hemorrhage
Source: Front Physiol. 2016 Aug 3;7:330. doi: 10.3389/fphys.2016.00330 (PMC4971438; doi:10.3389/fphys.2016.00330)
Supplement: Supplementary file 1 [file DataSheet1.DOCX]

# **Supplementary material**

**Neuroanatomical description**

From the hematoxylin and eosin (HE) and peroxidase activity of hemoglobin (PO) staining a number of subventricular and periventricular regions of interests (ROI) were chosen. Neuroanatomically defined ROI´s were located in the rostral forebrain (Level 1), caudal forebrain (Level 2), rostral midbrain (Level 3) and caudal midbrain (Level 4).

The following neuroanatomical regions were investigated.

ROI´s at Level 1 include the rostral lateral ventricle, cortex cerebri, corpus callosum, and nucleus caudatus.

ROI´s at Level 2, similarly, include the medial lateral ventricle, cortex cerebri, corpus callosum, and nucleus caudatus.

ROI´s at Level 3, include the caudal lateral and dorsal ventricle (ventricularis lateralis), cortex cerebri, corpus callosum, nucleus caudatus, fimbria fornicis, choroid plexus, and rostral portion of the hippocampus and pre-thalamic areas.

ROI´s at Level 4 include the third (hypothalamic portion) and dorsal ventricle (ventricularis lateralis), cortex cerebri, corpus callosum, nucleus caudatus, fimbria hippocampi, the hippocampus, dorsal thalamic nuclei, nucleus habenula, choroid plexus and the subfornical organ.

The definition of neuroanatomy and nomenclature used follows that described in the “Atlas of the rabbit brain and spinal cord”, by Shek et al. (Shek JW 1986).

**Figure Legends**

**Supplementary Figure 1. Hb immunolabeling within the brain of preterm control (non-IVH) rabbit pups.** Fluorescence microscope analyses of Hb immunofluorescence labeled (as described in Figure 4), control (non-IVH) animals. Images illustrate the same regions and levels of the brain as in Figure 4. Control animals showed only a few labelled (displayed in **red**) erythrocytes in the brain (arrows) and no Hb immunolabeling of extracellular free Hb, as compared with the widespread distribution and high amounts detected in IVH-animals (Figure 4). DAPI labelled cells are **blue**. Scale bar of slide scan images indicates 2.5 mm and of ROI images indicate 25 µm.

# **References**

1. Shek JW, Wen GY, Wisniewski HM. Karger, Basel. 1986, ISBN 3-8055-3814-6
