# Supplementary figures and images for: High Presence of Extracellular Hemoglobin in the Periventricular White Matter Following Preterm Intraventricular Hemorrhage
Source: Front Physiol. 2016 Aug 3;7:330. doi: 10.3389/fphys.2016.00330 (PMC4971438; doi:10.3389/fphys.2016.00330)

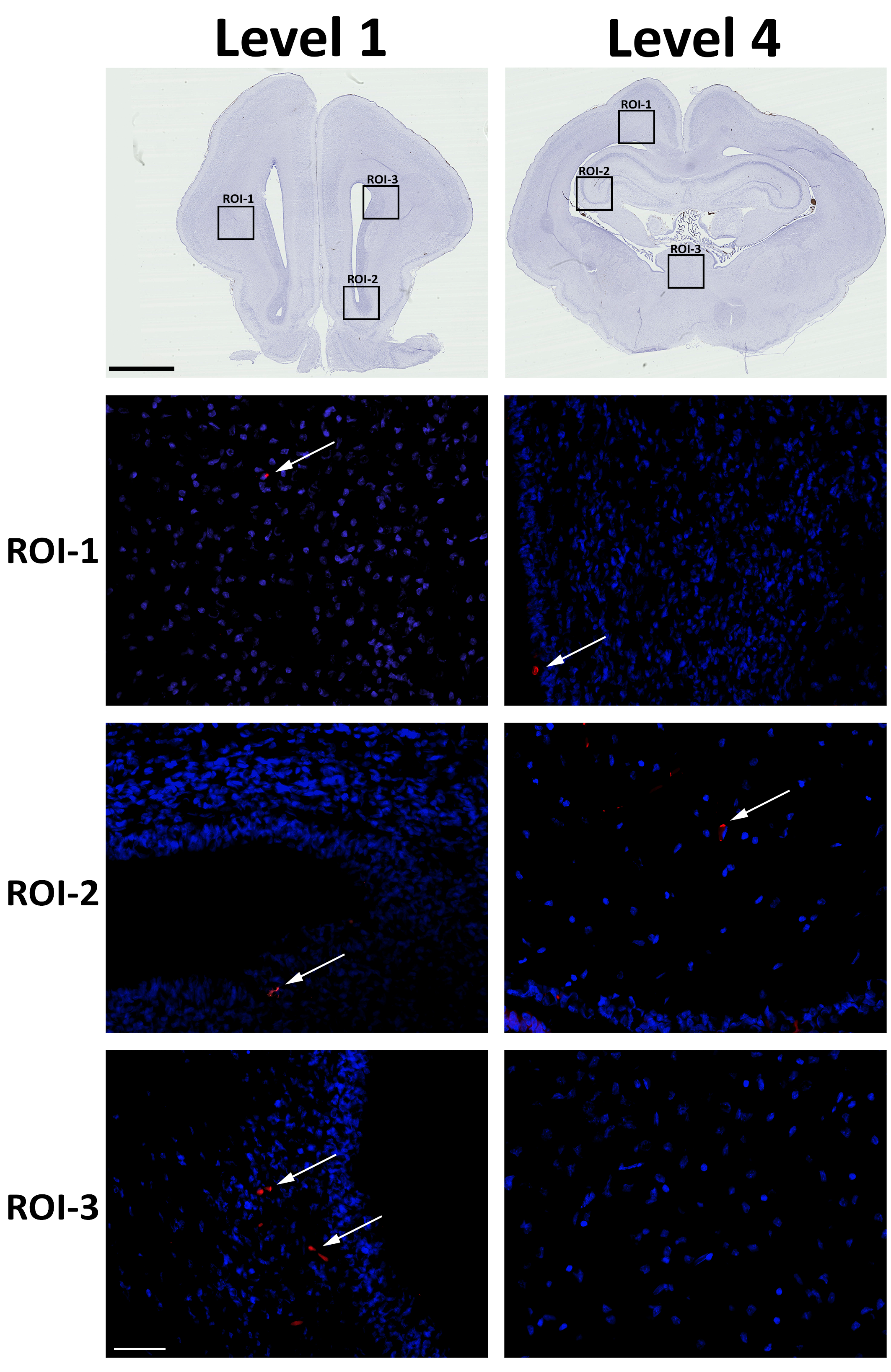

Supplement: Supplementary file 2 [file Image1.TIF]
